# The pediatric common variable immunodeficiency — from genetics to therapy: a review

**DOI:** 10.1007/s00431-021-04287-6

**Published:** 2021-12-23

**Authors:** Aleksandra Szczawinska-Poplonyk, Eyal Schwartzmann, Ewelina Bukowska-Olech, Michal Biernat, Stanislaw Gattner, Tomasz Korobacz, Filip Nowicki, Monika Wiczuk-Wiczewska

**Affiliations:** 1 Department of Pediatric Pneumonology, Allergy and Clinical Immunology, Institute of Pediatrics, Poznan University of Medical Sciences, Karol Jonscher University Hospital, 27/33 Szpitalna Street, 60-572 Poznan, Poland; 2 Department of Medical Genetics, Poznan University of Medical Sciences, 8 Rokietnicka Street, 60-806 Poznan, Poland

**Keywords:** Common variable immunodeficiency, Children, Genetics, Therapy, Infections, Autoimmunity

## Abstract

Common variable immunodeficiency (CVID) is the most prevalent antibody deficiency, characterized by remarkable genetic, immunological, and clinical heterogeneity. The diagnosis of pediatric CVID is challenging due to the immaturity of the immune response and sustained actively developing antibody affinity to antigens and immunological memory that may overlap with the inborn error of immunity. Significant progress has been recently done in the field of immunogenetics, yet a paucity of experimental and clinical studies on different systemic manifestations and immunological features of CVID in children may contribute to a delayed diagnosis and therapy. In this review, we aimed at defining the variable epidemiological, etiological, and clinical aspects of pediatric CVID with special emphasis on predominating infectious and non-infectious phenotypes in affected children.

*Conclusion*: While pediatric CVID is a multifaceted and notorious disease, increasing the pediatricians’ awareness of this disease entity and preventing the diagnostic and therapeutic delay are needed, thereby improving the prognosis and survival of pediatric CVID patients.

**Table:** 

**What is Known:** *• CVID is an umbrella diagnosis characterized by complex pathophysiology with an antibody deficiency as a common denominator*. *• It is a multifaceted disease characterized by marked genetic, immunological, and clinical heterogeneity.*.	
**What is New:** *• The diagnosis of pediatric CVID is challenging due to the immaturity of innate and adaptive immune response*. *• Increasing the pediatricians’ awareness of CVID for the early disease recognition, timely therapeutic intervention, and improving the prognosis is needed*.

**What is Known:**

*• CVID is an umbrella diagnosis characterized by complex pathophysiology with an antibody deficiency as a common denominator*.

*• It is a multifaceted disease characterized by marked genetic, immunological, and clinical heterogeneity.*.

**What is New:**

*• The diagnosis of pediatric CVID is challenging due to the immaturity of innate and adaptive immune response*.

*• Increasing the pediatricians’ awareness of CVID for the early disease recognition, timely therapeutic intervention, and improving the prognosis is needed*.

## Introduction

Common variable immunodeficiency (CVID) belongs to a phenotypically and immunologically heterogeneous and complex group of primary immunodeficiencies (PIDs). Primary antibody deficiencies (PADs) constitute the most prevalent and numerous categories of inborn errors of immunity, and CVID is the most common symptomatic hypogammaglobulinemia. This condition is notorious for its unfortunate and severe associated outcomes as infections, autoimmunity, granulomatous disease, organ-specific immunopathology, and malignancy. Moreover, the psychological and social burden of this chronic and incurable disease is causing a remarkable mental deterioration in affected patients.

While CVID is an intrinsic impairment of antibody production, the process of diagnosing CVID in children is challenging as no single clinical feature or laboratory test can establish the diagnosis. Among pediatric patients, antibody deficiency is quite common and may mirror the transient immune immaturity of B lymph cell functions, such as immunoglobulin class switch recombination (CSR), somatic hypermutation (SHM), and affinity maturation to antigens [1]. Exclusion of other primary antibody deficiencies and also secondary causes of hypogammaglobulinemia, which comprise a vast list of multifactorial etiologies, including various external influences, such as malnutrition, infections, systemic diseases, malignancies, and immunosuppressive therapy, is an important part of the definitive diagnosis of CVID [2]. Attempts are being made to identify prognosis, patterns, and severity scale of outcomes among pediatric-onset and adult-onset CVID patients [3, 4]. According to the 2014 European Society for Immunodeficiencies (ESID) revised diagnostic criteria [5], CVID has been suggested to be diagnosed after the age of 4, which significantly influences the prevalence of the disease in the pediatric population. This criterion of age has also been adopted for the purpose of excluding overlapping diagnoses of antibody deficiencies in early childhood, such as transient hypogammaglobulinemia of infancy (THI) [6] and unclassified hypogammaglobulinemia, and consequently, improving the specificity of the pediatric CVID diagnosis. 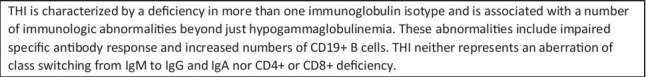


Importantly, those revised criteria also recommend to rule out profound T lymph cell deficiencies and clearly define the minimal age-matched T cell absolute count to preclude severe combined and combined immunodeficiencies (SCID and CID, respectively), such as CD40 ligand (CD40L) [7] and serine-threonine-kinase-4 (STK4) [8] deficiencies or the immunodeficiency, centromeric instability, and facial anomalies syndrome (ICF) [9]. These immunodeficiencies most frequently manifest early in the child’s life mimicking CVID and later develop the phenotype of CID in which gradual T cell depletion occurs. The particular phenotype of CD27 deficiency may also present as CVID but represents a cellular deficiency with immune dysregulation and Epstein-Barr virus (EBV)-driven hemophagocytic lymphohistiocytosis (HLH) and lymphoproliferation [10].
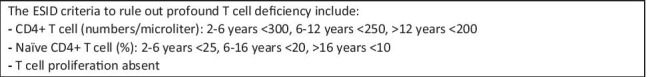


Other points of ESID diagnostic criteria may be, however, debatable in relation to young pediatric patients, as generation of switched memory B cells and immune response to vaccine recall antigens may be poor and diagnostically misleading.
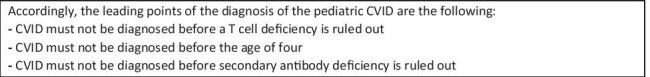


The following review was conducted to gather, resume, and conclude the data concerning the pediatric CVID and to highlight differences in the age of onset of patients in multiple parameters. It was also aimed at increasing awareness of this disease entity among pediatricians to prevent diagnostic delay and facilitate the implementation of specialized treatment options.

## Epidemiology

The epidemiological tendencies of pediatric CVID are difficult to be estimated precisely due to both age-related and geographic disparities which presumably determine further essential factors, such as availability of PID-centered medical facilities, pediatricians’ awareness of CVID, and access to the data of PID registries. The estimated overall prevalence of the disease varies from 1:10 000 to 1:100 000, with the highest CVID proportion among all PIDs reported in the USA (40.2%) and the lowest rates observed in the Middle East (2.6%) and Africa (1.3%) [11]. The observed striking discrepancies between regional CVID distributions are closely related to the country’s medical progress, which is in turn determined by its socio-economic status. The CVID prevalence has been correlated with the Human Development Index (HDI) of the United Nations Development Program (UNDP) and proved to be the highest in developed countries with high HDI and the best health care capability where immunodeficiencies are systematically monitored in registries [12].

Within the United States Immunodeficiency Network (USIDNET) report, CVID patients were stratified based on the age of diagnosis, and among all CVID cases, pediatric patients with either early (2–10 years of age) or adolescent (11–17 years of age) onsets amounted to as many as 42% of this cohort (24% and 18%, respectively) [4]. Interestingly, a 10-year observational study on an international large cohort of patients diagnosed with CVID has shown an interesting shift from male predominance in childhood to female predominance in adulthood, pointing to possible differences in genetic and environmental impact on the age-dependent epidemiology [13]. On the European background, data originating from the German National Registry of PIDs [14] pointed to the pediatric-onset of CVID in 35% of affected individuals, while in half of them, the predominating onset age of presenting symptoms was between 1 and 5 years.

The investigation of epidemiological aspects of the most frequent comorbidities in pediatric CVID [4] revealed that while sinopulmonary infections were the most frequently experienced disorders across all age groups, otitis media affected children nearly twice as frequently as adults (60.6% versus 33.3% of all CVID patients). Moreover, non-infectious complications, such as failure to thrive and developmental delay, were more common in the CVID-affected children [4]. Further correlations between the time of onset and the spectrum of clinical manifestations have also been documented [5], indicating a greater risk of developing autoimmune hematological disorders in pediatric CVID onset and an inverse correlation between the onset age and the risk of malignancy. CVID was also showed to significantly influence the patient’s life expectancy. The retrospective analysis of CVID patients reported to the ESID registry showed their considerably reduced lifespan compared to that of the general population [15]. Whereas the mortality in CVID was registered in patients aged from 6 to 84 years and was calculated to be 3,8%, the death rate was inversely proportional to the age and was the highest in children from 5 to 14 years old. Accordingly, the Years of Life Lost (YLL) factor was 22 times higher in this pediatric CVID cohort than in the general population. An increased risk of premature death was associated with the diagnostic delay, a relevant predictive factor reflecting the degree of healthcare system efficiency and CVID awareness among physicians. It is also worth noting that the mortality rate given in this report was four times higher in CVID patients with parental consanguinity, thereby supporting the hypothesis of monogenic, autosomal recessive disease underlying the CVID diagnosis in this group [15, 16].

## The genetic and epigenetic background of CVID

The genetic etiology of CVID reflects complex processes of B cell antigen signaling, activation, survival, migration, and maturation to generate terminal stages of switched memory B cells and plasma cells. The B cell developmental impairment and hypogammaglobulinemia may result from genetic defects of many receptors and ligands, activating co-stimulatory molecules and intracellular signaling molecules. Furthermore, mutations of genes linked to antibody production defects and immune dysregulation with autoimmunity, lymphoproliferation, enteropathy, splenomegaly, and granulomatosis have been identified thus far in a proportion of affected patients [17]. Genes that have been identified in monogenic CVID on the European background include *ICOS* (inducible T cell co-stimulator), *TNFRSF13B* (transmembrane activator and calcium modulator and cyclophilin ligand interactor, TACI), *TNFRSF13C* (B cell-activating factor belonging to the tumor necrosis factor (TNF) family, BAFF-receptor, BAFF-R), *TNFSF12* (TNF-like weak inducer of apoptosis, TWEAK), *CD19*, *CD81*, *CR2* (CD21), *MS4A1* (*membrane-spanning 4A1*, CD20), *TNFRSF7* (CD27), *IL21*, *IL21R*, *LRBA* (lipopolysaccharide (LPS)-responsive beige-like anchor protein), *CTLA4* (cytotoxic T lymphocyte-associated antigen 4), *PRKCD* (protein kinase C delta), *PLCG2* (phospholipase C gamma 2), *NFKB1* (nuclear factor kappa B1), *NFKB2* (nuclear factor kappa B2), *PIK3CD* (phosphoinositide 3-kinase (PI3K) catalytic subunit delta), *PIK3R1* (phosphoinositide 3-kinase (PI3K) regulatory subunit 1), *VAV1* (Vav guanine nucleotide exchange factor 1), *RAC2* (Rac family small GTPase 2), *BLK* (B-lymphoid tyrosine kinase), *IKZF1* (IKAROS), and *IRF2BP2* (interferon regulatory factor 2 binding protein 2) [17]. The expanding spectrum of genes involved in clinical and immunological phenotypes implicates that CVID is an umbrella diagnosis. Additionally, CVID shows high prevalence among all PIDs, but despite recent advances in genomics [18–20], their overall diagnostic rate remains low, with pathogenic gene variants identifiable in a limited proportion of patients, ranging from merely 2–10% [17] up to 54% in populations with a high rate of consanguinity [21]. The current genetic landscape of CVID and CVID-like disorders with their predominating clinical phenotypes is displayed in Table 1.

**Table 1:** Genes associated with CVID and CVID-like disorders and their clinical phenotypes

			Current genetics of CVID and CVID-like disorders				
Infections		Autoimmunity	Atopy	Malignancy	EBV, HLH	CID / SCID	Syndromic
*AIDCA*	*IL21*	*AIDCA*	*CTLA4*	*BAFFR*	*CARD11*	*CD27*	*DNTMT3B*
*BACH2*	*IL21R*	*BACH2*	*DOCK8*	*CD27*	*CD27*	*CD40L*	*KMT2D*
*BAFFR*	*IRF2BP2*	*BAFFR*	*FOXP3*	*CD70*	*CD70*	*CD70*	*LIG4*
*BLK*	*KMT2D*	*CD19*	*LRBA*	*CXCR4*	*CTLA4*	*DCK1*	*RTEL1*
*BLNK*	*LIG1*	*CD81*	*PLCG2*	*DCK1*	*GATA2*	*DCLRE1C*	*SPINK5*
*BTK*	*LIG4*	*CTLA4*	*RAC2*	*DCLRE1C*	*IKBKG*	*DNTMT3B*	*ZBTB24*
*CARD11*	*LRBA*	*FOXP3*	*SPINK5*	*DOCK8*	*IL2RG*	*DOCK8*	
*CD19*	*NFKB1*	*ICOS*		*FOXP3*	*ITK*	*IKBKB*	
*CD20*	*NFKB2*	*IKZF1*		*GATA2*	*LRBA*	*IL2RG*	
*CD21*	*PIK3CD*	*IL12RB1*		*ICOS*	*PIK3CD*	*LIG1*	
*CD27*	*PIK3R1*	*IL21*		*IKZF1*	*PIK3R1*	*LIG4*	
*CD40L*	*PLCG2*	*IL21R*		*IL12RB1*	*SH2D1A*	*RAG 1 / 2*	
*CD70*	*PRKCD*	*IRF2BP2*		*LIG4*	*STAT3*	*RFX5*	
*CD81*	*RAC2*	*KMT2D*		*NFKB1*	*STXBP2*	*RTEL1*	
*CTLA4*	*RAG 1 / 2*	*LRBA*		*PIK3CD*	*XIAP*	*SPINK5*	
*CTNNBL1*	*RFX5*	*NFKB1*		*PIK3R1*		*WAS*	
*CXCR4*	*RFXANK*	*NFKB2*		*PMS2*		*ZBTB24*	
*DCK1*	*RTEL1*	*PIK3CD*		*PTPRC*			
*DCLRE1C*	*SH2D1A*	*PIK3R1*		*RNF31*			
*DNMT3B*	*SPINK5*	*PLCG2*		*TACI*			
*DOCK8*	*STAT3*	*PRKCD*		*TINF2*			
*GATA2*	*TACI*	*PTPRC*		*WAS*			
*ICOS*	*TCF3*	*RAC2*					
*IGHM*	*THBD*	*RAG 1 / 2*					
*IKBKB*	*TWEAK*	*STAT3*					
*IKBKG*	*UNC93B1*	*TACI*					
*IKZF1*	*VAV1*	*TCF3*					
*IL12RB1*	*WAS*	*TWEAK*					
*IL12RB1*	*ZBTB24*						
*IL2RG*							

Despite that the main tool for diagnosis of CVID remains clinical, it is highly recommended to obtain a genetic workup and molecular analysis in all subjects with unclear and severe clinical phenotype [22]. Nevertheless, most patients with a diagnosis of CVID do not follow a classical Mendelian pattern of inheritance, often representing single sporadic cases. It has been suggested that beyond the monogenic model of inheritance, another explanation of CVID origin is multifactorial, digenic, or polygenic, and alternatively, that accumulation of rare functional variants, somatic mutations, or epigenetic phenomena [18, 19, 23] may show a causal relationship with the regulation of B cell development and functions.

These observations could guide further investigations, and epigenetics may, therefore, contribute to explaining the pathogenesis of CVID in patients who lack a molecular genetic diagnosis. Histone and chromatin modifications or differences in DNA methylation level have been shown in switched and non-switched memory B cells in some CVID patients. Unusual hypermethylation of B cell development and function-relevant genes, such as *PIK3CD*, *BCL2L1* (Bcl-2-like 1), *RPS6KB2* (ribosomal protein S6 kinase beta 2), *TCF3* (transcription factor 3), or *KCNN4* (potassium calcium activate channel subfamily N member 4) and abnormal demethylation during the transition from naïve to memory B cells [24, 25]. Non-coding RNA molecules, transcribed from DNA and not translated into proteins, exert their regulatory effects on gene expression and protein translation by influencing DNA transcription and mRNA post-transcriptional changes. In particular, microRNAs (miRNAs) have critical regulatory functions in cell differentiation and proliferation, thereby being involved in B and T lymph cell development and function [26, 27]. Multiple miRNAs, such as miRNA-155, miRNA-181b, miRNA-351, and miRNA-210, are implicated in the regulation of B cell function, germinal center formation, and antibody response on the antigenic challenge [28].

## The immune system in pediatric CVID

Whereas CVID is perceived as a clinically heterogeneous group of disorders with complex genotype–phenotype mutual relationships, antibody deficiency is their common denominator as well as a constant and essential diagnostic criterion of CVID. Hypogammaglobulinemia in CVID is diagnosed as a marked decrease of serum IgG and IgA with or without low serum IgM levels. In children, due to the diverse dynamics of immunoglobulin isotypes that change with age, levels lower than two standard deviations below age-matched normal values are measured at least twice to support the diagnosis. The 2019 ESID Registry-working definitions [29] are based on clinical and immunological criteria unified with revised 2014 ESID guidelines on the diagnosis of CVID. Along with hypogammaglobulinemia, the absence of antigen-specific antibodies expressed as poor antibody response to vaccines and the low relative values of CD19 + CD27 + IgD-switched memory B cells (< 70% of age-related normal values) are the next crucial items of this definition.

However, understanding the immunopathogenesis of CVID in childhood requires a deep insight into the developmental processes of the B and T cell subset compositions and their functions. The investigation of the alterations within the B cell compartment by the flow cytometric immunophenotyping showed remarkable age-related shifts, with gradual loss of B cell naїvete and development of B cell memory [30]. Accordingly, stratifications used in adult patients with CVID, namely EUROclass, Freiburg, and Paris classifications [31], based primarily on reduced numbers of switched memory B cells cannot be directly extrapolated to pediatric patients with CVID due to the ongoing maturation of the immune specificity to antigens in children [1, 32].

Abnormalities of various pathways across the adaptive and innate immune responses have been revealed in pediatric CVID, and extensive attempts have been undertaken to establish correlations between the interrupted immunological homeostasis and clinical complications. The most frequently reported abnormalities within the B cell compartment were a defective generation of total memory B cell population [33–35], reduced number of switched memory B cells, reflecting impairment in germinal center reaction [34], deficiency in CD3 + CD4 + CD45RO + CD185 + (CXCR5 +) follicular T helper cell, and consequently, inefficient CSR, SHM, and immunoglobulin affinity maturation [36], yet defective pre-germinal center B cell maturation pathways have also been shown in CVID [37]. Among T cells, most frequently, low numbers of total CD4 + T helper cell subsets, essential for effective B cell response and antibody production [33, 35], followed by deficiency of CD3 + CD4 + CD45RA + naïve T cells and CD3 + CD4 + CD45RA + CD31 + recent thymic emigrants were reported in pediatric CVID [33]. Deficiencies of T CD4 + cells were accompanied by an increase in CD3 + CD8 + CD45RO + cytotoxic memory T cells [33] and along with alterations within B cell subsets foremostly correlated with infectious complications of the respiratory tract and chronic diarrhea. It has been hypothesized that a CD3 + CD4 + CD25 + Foxp3 + regulatory T cell (Treg) dysfunction and its association with increased numbers of CD19 + CD38 low CD21 low immature activated B cells may be involved in impaired immune tolerance in CVID and contribute to the immunopathology of autoimmune phenomena and immune dysregulation [38]. However, in children, the aberrant Treg-dependent tolerogenic pathways have not been shown, and in pediatric CVID patients, the more severe disease pattern has been ascribed to the skewed Th1 polarization and excessive C–C chemokine receptors CCR5 (CD195) and CCR7 (CD197) expression [39].

Bridging the innate and adaptive immune response pathways, the regulatory role of Toll-like receptors (TLRs) in CVID has been investigated, and the results pointed to an impaired TLR9-mediated signaling and IFN-α production as well as reduced generation of tumor necrosis factor (TNF)-α following induction of TLR4 expression [40]. In children with CVID, a depressed CD11a adhesion molecule on lymphocytes and neutrophils along with an increased CD18 expression, associated with decreased percentages and increased NK cell cytotoxicity, has been found [41]. In the light of the persistently aberrant adaptive immune response in CVID and defective clearance of pathogens, these findings suggest an innate immune system activation that may, in turn, predispose CVID-affected children to the development of chronic inflammatory complications. Further studies are needed to comprehend the interrelated functions of various lymph cell subsets and their potential predictive roles in autoimmunity, autoinflammation, lymphoproliferation, and organ-specific immunopathology in pediatric CVID.
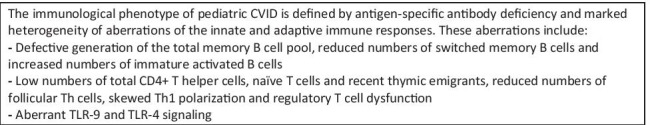


## Infections and infectious complications

In the era of increasing awareness of CVID in pediatric patients and appreciating the clinical heterogeneity of the disease, facing autoimmunity, autoinflammation, immune dysregulation, and lymphoproliferation, infections and infectious complications remain the leading cause of morbidity and mortality of children affected with CVID.

In a systematic review including a large cohort of CVID patients, infection pattern and frequency together with predominating immune response abnormalities have been analyzed [42]. The reported findings demonstrated that in CVID children, comparably to adults, pneumonia was the most prevalent infection, assessed in as much as 73% of pediatric patients, and it was followed by upper respiratory tract infections, such as otitis media, pharyngitis, and tonsillitis as occurring in 65%, and gastrointestinal infections in 44% of them. Infections caused by *Streptococcus pneumoniae*, *Haemophilus influenza*, *Staphylococcus aureus*, and *Pseudomonas aeruginosa* were associated with the severe course of the disease. In single patients, other severe infectious episodes, such as osteomyelitis [4, 13], septic arthritis [43], central nervous system (CNS) infections [43, 44], and sepsis [4, 13, 45], were reported. Bacterial etiologies were noted most frequently, in 41.7% of infectious episodes compared to viral, parasitic, or fungal infections, assessed in 25.4%, 18.8%, and 3.4%, respectively [42]. Interestingly, a higher rate of pneumonia was inversely proportional to the patients’ age, and as respiratory tract infections are the leading clinical indicator of pediatric CVID, they contributed significantly to an early diagnosis of the disease [42]. Pediatric patients who showed a lower percentage of the total T cell pool and an increased total B cell absolute count with a decreased switched memory B cell subset were more susceptible to the severe infectious phenotype. It has also been shown that CD4 + T lymphopenia was associated with a higher frequency of viral infections in this CVID cohort [42]. Among respiratory viruses, rhinovirus, respiratory syncytial virus, and adenovirus were the most frequent causes of respiratory exacerbations, impaired lung function, hospital admissions, and antibiotic therapy in pediatric CVID [46]. While IgG levels on immunoglobulin replacement therapy (IgRT) remained normal, low serum IgA levels were associated with the increased susceptibility to viral respiratory infections. It might be therefore assumed that other mechanisms of the local and systemic, innate and adaptive immune responses beyond IgG serum levels, play an essential protective role against viral infections, thereby explaining the limited effectiveness of IgRT in the light of the changing spectrum of respiratory pathogens [47].

Further long-term complications and unfavorable outcomes of respiratory infections include irreversible parenchymal and interstitial lung disease, lung fibrosis, and airway disease with bronchiectasis, which is the most common recognizable post-infectious complication in children with CVID [48]. Opportunistic infections of the respiratory tract caused by *Pneumocystis jiroveci* and *Mycobacterium tuberculosis* in CVID children are uncommon and indicate a deficiency in T and B lymphocytes [49]. The digestive system is also an important infection site in antibody-deficient children, in whom the spectrum of gastrointestinal pathologies comprises various autoimmune and lymphoproliferative disorders [50]. The most common infectious etiologies of chronic diarrhea episodes are *Campylobacter*, *Salmonella*, *Shigella*, and *Giardia lamblia*, whereas norovirus infections can lead to exacerbations of enteropathies, and consequently, intestinal villous atrophy, malabsorption syndrome, and failure to thrive with impaired physical development [49, 51].

Severe, systemic infections of viral and bacterial etiologies have also been reported in CVID pediatric patients. In case of the enteroviral disseminated, life-threatening disease manifested by fever, dermatomyositis, and systemic inflammatory reaction with progression to meningitis may develop. Infections with herpes viruses in immunodeficient children may pose a high risk of unfavorable course and long-term sequelae, such as encephalitis caused by herpes simplex virus (HSV)-1 infection [42, 52] or disseminated cytomegalovirus (CMV) infection with nephrotic syndrome [53].

## Non-infectious phenotypes of pediatric CVID

Whereas in children recurrent infections are the most common symptoms of CVID, inappropriate immunosurveillance, imbalanced biological lymph cell homeostasis, and skewed T and B cell response with reduced tolerogenic lymph cell pools are underpinning the wide spectrum of non-infectious phenotypes, such as autoimmune disorders, granulomatous diseases, and polyclonal lymphoproliferation which have been highlighted as hallmarks of this disease entity [5, 29].

Autoimmune diseases are the second manifestation of systemic or organ-specific immunopathology in CVID after infections, occurring in 10–30% of affected patients. In early-onset CVID, diagnosed before the age of 10 years, the young age negatively correlates with the risk of autoimmune complications [39]. Monogenic CVID characterized by impaired self-tolerance and an autoimmune phenotype include ICOS [54], LRBA [55, 56], CTLA-4 [57], NF-kappa B1, and NF-kappa B2 [17] deficiencies, associated with a strikingly high prevalence of a wide spectrum of autoimmune disorders ranging from 31 to 76% of pediatric CVID cases compared to 10.2% of CVID patients in the USIDNET Registry [58]. The mechanisms that have been postulated to be involved in the pathogenesis of autoimmunity are defective B cell tolerance, expansion of CD21low B cell subset, altered BAFF signaling, impaired generation of switched memory B cells, defective somatic hypermutations, and reduced generation of Tregs [59, 60]. The most prevalent autoimmune disorder in pediatric CVID is cytopenia — autoimmune thrombocytopenia (ITP), autoimmune hemolytic anemia (AIHA), Evans syndrome, and neutropenia, followed by multiple clinical diagnoses, such as autoimmune thyroiditis, polyarthritis, inflammatory bowel disease (IBD), celiac disease (CD), Sjogren syndrome, dermatomyositis, alopecia, and psoriasis [59, 61]. It has been noted that children who experience autoimmune diseases are also prone to develop other non-infectious complications of CVID, such as granulomatous disease, lymphoproliferation, and organ-specific immunopathology [58]. The CVID-related granulomatous disease has been reported in 8–20% of patients, with young adulthood being the most frequent age of its recognition, thereby, a limited number of reports on granulomatous lymphocytic interstitial lung disease (GLILD) in pediatric CVID are found in the literature [62]. Pulmonary involvement in the form of granulomatous interstitial lung disease (GLILD), a non-infectious lymphoproliferative disorder, encompasses a spectrum of distinct lung immunopathologies that include granulomatous disease, follicular bronchiolitis, interstitial pneumonia, and lymphoid hyperplasia, frequently accompanied by airway disorders, such as bronchiectasis and tree-in-bud pattern [63, 64]. It is considered a systemic disease, and extrapulmonary manifestations are its integral parts. The organ-specific immunological predictors of a diagnosis of GLILD in CVID-affected patients are splenomegaly and hypersplenism, autoimmune cytopenias, in particular AIHA and ITP, lymphadenopathy, enteropathy, autoimmune hepatitis, and polyarthritis. Whereas the lung is the most common organ affected by the granulomatous disease in pediatric CVID patients, granulomas can also be localized in the spleen, liver, intestine, kidneys, eyes, skin, parotid glands, and the central nervous system [65].

Immune dysregulation manifesting as asthma is also frequently observable in pediatric CVID, accountable for 31.2% of the chronic respiratory symptoms, according to the USIDNET registry [66], and it has been considered as the most important complication related to low IgA and IgM levels.

Polyclonal lymphoproliferation is observable in CVID patients as, but not limited to, peripheral and also pulmonary and abdominal lymphadenopathy, posing an increased risk of malignancy. The risk of lymphoid malignancies in pediatric-onset CVID has been estimated to 2,5% compared to 8.5% among those affected individuals, in whom CVID was diagnosed in adulthood and the overall prevalence of cancers in PIDs reaching 5.7%. Hence, malignancy is the leading cause of morbidity and mortality in CVID, and after infections, it is the second cause of death both among pediatric and adult patients [67]. In particular, in children affected with CVID, B cell non-Hodgkin lymphomas and low-grade astrocytoma have been noted [68, 69]. Importantly, those PID patients carry a lifetime risk of malignancies, not solely confined to the lymphatic origin, but also of stomach cancer in adulthood [65]. The postulated molecular mechanisms that have been implicated in malignant processes in CVID include defective recognition of malignant cells by the adaptive immune responses, selective accumulation of mutations in genes enabling to survive malfunctioning immune destruction, and mutations of genes involved in the cell cycle checkpoints [67, 68, 70]. This particular association of malignancy-related morbidity and mortality in pediatric CVID requires a special emphasis on the need for increased pediatricians’ awareness to improve survival and long-term prognosis for affected children.

## Preventive measures and therapeutic options for pediatric CVID

The pediatricians’ and primary care physicians’ awareness is a key to the timely undertaking prevention of infection in pediatric CVID patients.

### Vaccinations.

The optimal vaccination status of the primary antibody immunodeficient children plays a fundamental preventive role against infections and infectious complications in this targeted group of patients. However, an important issue is optimizing the vaccinations in these particularly infection-vulnerable children, and to assure their both immunogenicity and safety [71]. Important questions and concerns have been addressed regarding the vaccine use in immunocompromised children, their protective value and beneficial effect, safety of live attenuated vaccines, rationale for monitoring the vaccine-induced antigen-specific immune response, and ultimately, whether and how pediatricians and primary care physicians could contribute to improving the immunization status of immunodeficient children.

Importantly, in CVID, along with antibody production defect and a B cell dysfunction, variable quantitative and qualitative deficiencies of T cells, NK cells, and innate immune cells are observable. As these complex impairments of immune response in CVID patients may considerably vary, recommendations for the administration of both inactivated and live attenuated vaccines need to be considered individually.

In CVID, live attenuated vaccines, such as oral poliomyelitis vaccine (OPV), live attenuated influenza vaccine (LAIV), yellow fever, smallpox and live bacterial vaccines, e.g., *Salmonella typhi* (Ty21a), are contraindicated as they confer a risk of adverse effects following vaccination (AEFI) [72]. A risk-benefits ratio of live measles and varicella vaccine administration needs to be considered in those pediatric CVID patients who despite B cell deficiency are capable to preserve their T cell number > 500 cells/mcL CD4 + and > 200 cells/mcL CD8 + cells and function assessed as normal mitogen response [73]. Another issue is an indication to live attenuated measles and varicella vaccines in those CVID patients who have already received RT-Ig as they are ineffective due to vaccine neutralization and not recommended. Inactivated vaccines are considered safe and well tolerated in CVID, yet most affected patients are not capable to mount a protective antibody response following immunization. However, despite the uncertainty of their immune response, administration of an inactivated influenza vaccine as well as pneumococcal and meningococcal vaccines is strongly recommended due to low antigen-specific antibodies in immunoglobulin preparations and a high risk of morbidity in pediatric CVID patients [73, 74].

### Immunoglobulin replacement therapy (IgRT).

Either administered intravenously (IVIg) or subcutaneously (SCIg), it is the mainstay of management of CVID pediatric patients, foremostly targeted at providing antigen-specific antibodies. It has been demonstrated that in children with CVID, Ig-RT has led to the achievement of satisfactory IgG serum levels, reduction in the incidence of respiratory tract infections, hospitalization rates, and antibiotic use [75]. Whereas Ig-RT has been proved to limit the severity and incidence of infections and infection-related organ damage, its role in controlling autoimmune and inflammatory disorders in CVID has not been precisely documented. The clinical effect of Ig-RT may vary among children suffering from respiratory tract infections as the controlling of infection due to influenza virus, rhinovirus, or adenovirus etiology is not adequate. Bronchiectasis is the most frequent suppurative irreversible organ damage in pediatric CVID which persists despite regular Ig-RT as it is associated with greater immunoglobulin consumption and requires higher doses of IgG [76]. In CVID, gastrointestinal tract involvement manifests as chronic diarrhea, malabsorption with steatorrhea, and rectal bleeding. The impact of Ig-RT on CVID-related enteropathy is not clear and the poor response to the therapy may be explained by the lack of protective serum and mucosal IgA which is not replenished by Ig-RT [75, 77].

### Supplementary therapy.

In those children with CVID who despite adequate Ig-RT suffer from recurrent sinopulmonary infections posing the risk of chronic lung damage, additional antimicrobial prevention with the use of co-trimoxazole, amoxicillin, or azithromycin is recommended. Antibiotic prophylaxis covering the most frequent pathogens, such as *Streptococcus pneumoniae* and *Haemophilus influenza*, proved to be particularly beneficial for children with CVID to prevent bronchiectasis and if they develop, prophylactic antibiotics should be used to improve the outcome [76–78].

Granulomatous lymphocytic interstitial lung disease is a potentially devastating non-infectious lymphoproliferative complication of CVID associated with a restrictive impairment of lung function, progressive respiratory insufficiency, and shortened survival. Lack of consensus recommendations for the monitoring and management of this condition in children results in the use of the treatment regimens elaborated for adult patients. The proposed first-line therapy for CVID-related GLILD is systemic corticosteroids (GCS), followed by azathioprine, mycophenolate mofetil either combined with GCS or as monotherapy, and rituximab (a monoclonal antibody against CD20) as the second-line therapy. In single cases only, infliximab (a monoclonal antibody against TNF-α), cyclophosphamide, cyclosporine, methotrexate, and hydroxychloroquine alone or in combination with Ig-RT have also been reported with different degree of remission [62, 76, 79].

With the ever-increasing incidence of CVID-related monogenic defects, future perspectives on modern diagnosis and targeted treatment modalities are being developed and implemented. The novel treatment strategies are primarily targeted at the control of impaired B and T cell homeostasis with autoimmunity and lymphoproliferation. The expanding therapeutic armamentarium comprises antiproliferative drugs, e.g., rapamycin; monoclonal antibodies, such as tocilizumab (anti-IL-6R) and jakinibs; and a new class of Jak kinase inhibitors, e.g., ruxolitinib [80]. The use of these biological drugs paves a new way to individualized pathogenetically directed therapy for pediatric CVID.

## Concluding remarks

**Fig. 1:**
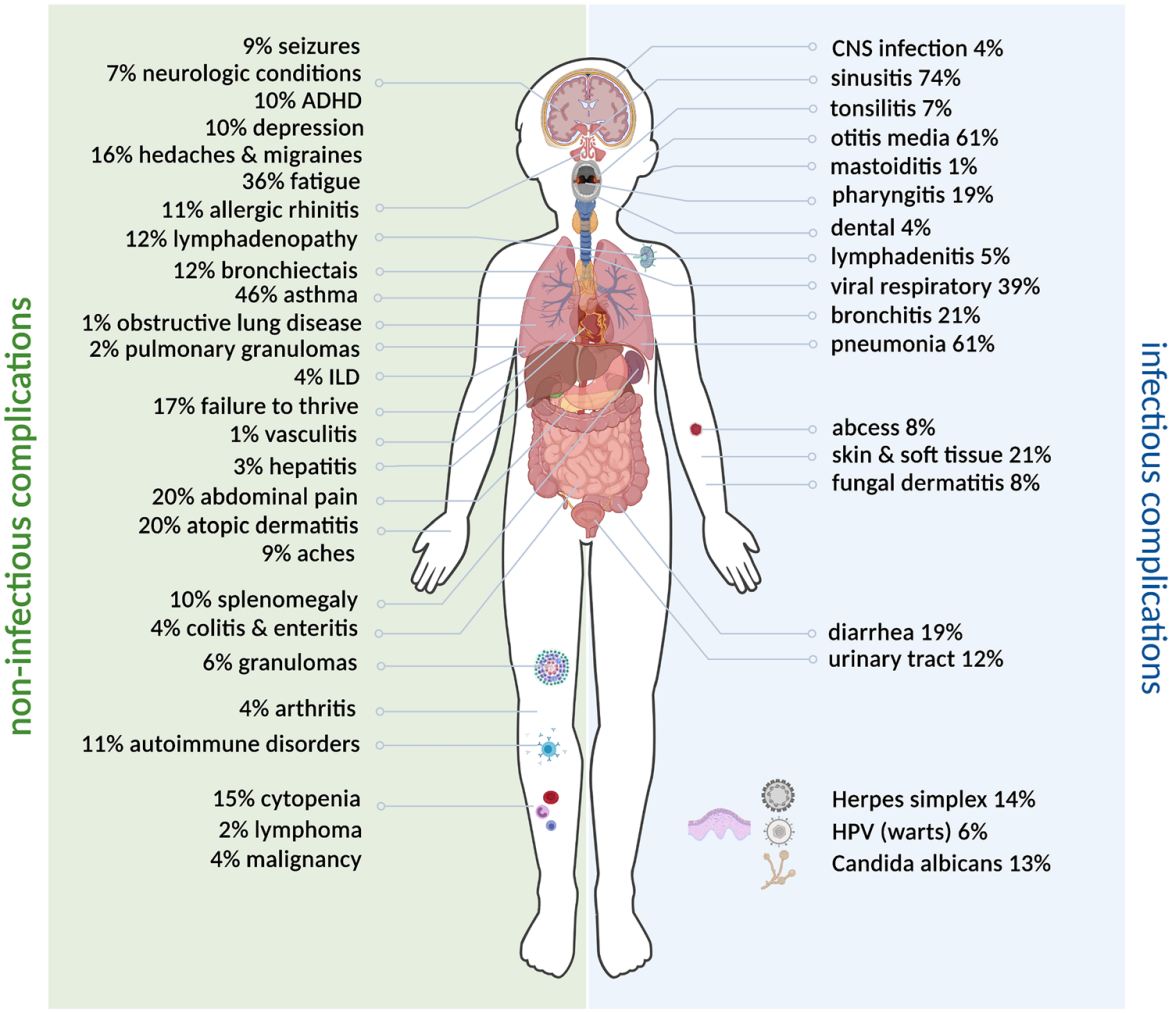
Stratification of infectious and non-infectious complications in pediatric CVID patients done using the USIDNET database and adopted from [4]

Whereas the genetic landscape of pediatric CVID is being expanded, it is no longer perceived as a spectrum of monogenic diseases, yet polygenic and environmental background, as well as epigenetic regulation of gene expression, has been postulated as relevant mechanism underpinning the immunopathogenesis of CVID. The diagnosis of CVID in children is challenging as the immaturity of the immune system with the defective generation of antigen-specific antibodies and immunological memory may overlap with an inborn error of immunity. The complexity of genotype–phenotype mutual relationships implicates a variety of clinical manifestations of CVID in children (Fig. 1). Pediatric CVID is a serious disease, foremostly burdened with chronic infections but non-infectious disorders, such as autoimmunity, lymphoproliferation, organ-specific immunopathology, and malignancy may also occur. Importantly, pediatric CVID shows its own age-related immunological and clinical specificity, and therefore, diagnostic and therapeutic guidelines targeted to adults cannot be merely extrapolated to children. Future investigations of the immunopathogenesis of pediatric CVID in the light of the dynamic development of the immune system and clinical studies are needed to better delineate this disease entity in children. Increasing the pediatricians’ awareness to tackling the diagnostic and therapeutic delay to improve the prognosis for CVID affected children is of paramount importance.
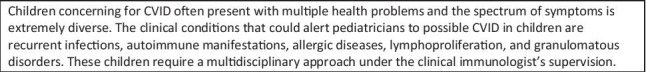


## Abbreviations

ADHD: Attention deficit/hyperactivity disorder
AEFI: Adverse effect following vaccination
AIDCA: Activation-induced cytidine deaminase
AIHA: Autoimmune hemolytic anemia
BACH: BTB domain and CNC homolog
BAFF: B cell-activating factor belonging to the tumor necrosis factor (TNF) family
BLK: B-lymphoid tyrosine kinase
BLNK: B-cell linker
BTK: Bruton tyrosine kinase
CARD: Caspase recruitment domain family member
CCR: CC-chemokine receptor
CID: Combined immunodeficiency
CMV: Cytomegalovirus
CSR: Class-switch recombination
CTLA: Cytotoxic T lymphocyte-associated antigen
CTNNBL1: Catenin beta-like
CVID: Common variable immunodeficiency
CXCR: CXC-chemokine receptor
DCK: Dyskerin
DCLRE: DNA cross-link repair
DNMT: DNA methyltransferase
DOCK: Dedicator of cytokinesis
EBV: Epstein-Barr virus
ESID: European Society for Immunodeficiencies
FOXP: Forkhead box protein
GATA: GATA binding protein
GLILD: Granulomatous lymphocytic interstitial lung disease
HDI: Human Development Index
HLH: Hemophagocytic lymphohistiocytosis
HSV: Herpes simplex virus
IBD: Inflammatory bowel disease
ICF: Immunodeficiency-centromeric instability-facial anomalies
ICOS: Inducible T cell co-stimulator
IFN: Interferon
IGHM: Immunoglobulin heavy constant µ
IgRT: Immunoglobulin replacement therapy
IKBKB: Inhibitor of nuclear factor κ B (NFκB) kinase subunit beta
IKBKG: Inhibitor of nuclear factor κ B (NFκB) kinase subunit gamma
IKZF: Ikaros zinc finger
IL: Interleukin
ILD: Interstitial lung disease
IRF2BP: Interferon regulatory factor 2 binding protein
ITK: Interleukin 2-inducible T-cell kinase
ITP: Immune thrombocytopenia
IVIg: Intravenous immunoglobulin
KCNN: Potassium calcium activate channel subfamily N member
KMT: Lysine methyltransferase
LAIV: Live attenuated influenza vaccine
LIG: Ligase
LRBA: Lipopolysaccharide (LPS)-responsive beige-like anchor protein
MiRNA: MicroRNA
MS: Membrane-spanning
NFKB: Nuclear factor kappa B
PAD: Primary antibody deficiency
PID: Primary immunodeficiency
PIK3CD: Phosphoinositide 3-kinase (PI3K) catalytic subunit delta
PIK3R: Phosphoinositide 3-kinase (PI3K) regulatory subunit
PLCG: Phospholipase C gamma
PMS: MS1 homolog
PRKCD: Protein kinase C delta
PTPRC: Protein tyrosine phosphatase receptor type C
RAC: Ras-related C3 botulinum toxin substrate
RAG: Recombination activating gene
RFX: Regulatory factor X
RFXANK: Regulatory factor X-associated ankyrin-containing protein
RNF: Ring finger protein
RPS6KB: Ribosomal protein S6 kinase beta
RTEL: Regulator of telomere elongation
SCID: Severe combined immunodeficiency
SCIg: Subcutaneous immunoglobulin
SH2D: Src homology 2 domain containing
SHM: Somatic hypermutations
SPINK: Serine peptidase inhibitor Kazal type
STAT: Signal transducer and activator of transcription
STXBP: Syntaxin binding protein
TACI: Transmembrane activator and calcium modulator and cyclophilin ligand interactor
TCF: Transcription factor
THBD: Thrombomodulin
THI: Transient hypogammaglobulinemia of infancy
TINF: TERF1-interacting nuclear factor
TLR: Toll-like receptor
TNF: Tumor necrosis factor
TNFRSF: Tumor necrosis factor receptor superfamily factor
TWEAK: TNF-like weak inducer of apoptosis
UNDP: United Nations Development Program
USIDNET: United States Immunodeficiency Network
WAS: WASP actin nucleation promoting factor
XIAP: X-linked inhibitor of apoptosis
ZBTB: Zinc finger and BTB domain containing

## References

[1] Szczawinska-Poplonyk A, Tapolska-Jozwiak K, Samara H (2016). The B cell compartment in antibody-deficient infants and young children – developing common variable immunodeficiency or transient immune maturation?. Ital J Pediatr.

[2] Patel SY, Carbone J, Jolles S (2019). The expanding field of secondary antibody deficiency: causes, diagnosis, and management. Front Immunol.

[3] Baloh C, Reddy A, Henson M, Prince K, Buckley R, Lugar P (2019). 30-year review of pediatric- and adult-onset CVID: clinical correlates and prognostic indicators. J Clin Immunol.

[4] Sanchez LA, Maggadottir MS, Pantell Ms, Lugar P, Cunningham-Rundles C, Sullivan K (2017) Two sides of the same coin: pediatric-onset and adult-onset common variable immunodeficiency. J Clin Immunol 37:592–602. 10.1007/s10875-017-0415-5 10.1007/s10875-017-0415-5 28755066

[5] Ameratunga R, Brewerton M, Slade C, Jordan A, Gillis D, Steele R (2014). Comparison of diagnostic criteria for common variable immunodeficiency disorder. Front Immunol.

[6] Dorsey MJ, Orange JS (2006). Impaired specific antibody response and increased B-cell population in transient hypogammaglobulinemia of infancy. Ann Allergy Asthma Immunol.

[7] Leite LFB, Maximo TA, Mosca T, Forte WCN (2020). CD40 ligand deficiency. Allergol Immunopathol.

[8] Cagdas D, Halacli SO, Tan C, Esenboga S, Karaatmaca B, Cetinkaya PG (2021). Diversity in STK4 deficiency and review of the literature. J Allergy Clin Immunol Pract.

[9] Von Bernuth H, Ravindran E, Du H, Frohler S, Strehl K, Kramer N (2014). Combined immunodeficiency develops with age in immunodeficiency-centromeric instability-facial anomalies syndrome 2 (ICF2). Orphanet J Rare Dis.

[10] Ghosh S, Bal SK, Edwards ESJ, Pillay B, Jimenez Heredia R, Erol Cipe F (2020). Extended clinical and immunological phenotype and transplant outcome in CD27 and CD70 deficiency. Blood.

[11] Modell V, Orange JS, Quinn J, Modell F (2018). Global report on primary immunodeficiencies: 2018 update from the Jeffrey Modell Centers Network on disease classification, regional trends, treatment modalities, and physician reported outcomes. Immunol Res.

[12] Weifenbach N, Schneckenburger AAC, Lotters S (2020). Global distribution of common variable immunodeficiency (CVID) in the light of the UNDP Human Development Index (HDI): a preliminary perspective of a rare disease. J Immunol Res.

[13] Janssen LMA, van der Flier M, de Vries E (2021) Lessons learned from the clinical presentation of common variable immunodeficiency disorders: a systematic review and meta-analysis. Front Immunol 620709. 10.3389/fimmu.2021.620709 10.3389/fimmu.2021.620709 PMC8021796 33833753

[14] El-Helou SM, Biegener AK, Bode S, Ehl SR, Heeg M, Maccari ME (2019). The German National Registry of primary immunodeficiencies (2012–2017). Front Immunol.

[15] Odnoletkova I, Kindle G, Quinti I, Grimbacher B, Knerr V, Gathmann B et al The burden of common variable immunodeficiency disorders: a retrospective analysis of the European Society for Immunodeficiency (ESID) registry data. Orphanet J Rare Dis 2018;13:201. 10.1186/s13023-018-0941-0 10.1186/s13023-018-0941-0 PMC6233554 30419968

[16] Mahlaoui N, Warnatz K, Jones A, Workman S, Cant A (2017). Advances in the care of primary immunodeficiencies (PIDs): from birth to adulthood. J Clin Immunol.

[17] Bogaert DJA, Dullaers M, Lambrecht BN, Vermaelen KY, De Baere E, Haerynck F (2016). Genes associated with common variable immunodeficiency: one diagnosis to rule them all?. J Med Genet.

[18] De Valles-Ibanez G, Esteve-Sole A, Piquer M, Gonzalez-Navarro EA, Hernandez-Rodriguez J, Laayouni H (2018). Evaluating the genetics of common variable immunodeficiency: monogenetic model and beyond. Front Immunol.

[19] Edwards ESJ, Bosco JJ, Ojaimi S, O’Heir RE, van Zelm MC (2021). Beyond monogenic rare variants: tackling the low rate of genetic diagnoses in predominantly antibody deficiency. Cell Mol Immunol.

[20] Ramirez NJ, Posadas-Cantera S, Caballero-Oteyza A, Camacho-Ordonez N, Grimbacher B (2021). There is no gene for CVID – novel monogenetic causes for primary antibody deficiency. Curr Opin Immunol.

[21] Abolhassani H, Hammarstrom L, Cunningham-Rundles C (2020). Current genetic landscape in common variable immunodeficiency. Blood.

[22] Ameratunga R, Lehnert K, Woon ST (2019). All patients with common variable immunodeficiency disorders (CVID) should be routinely offered diagnostic genetic testing. Front Immunol.

[23] Aggarval V, Banday AZ, Jindal AK, Das J, Rawat A (2020). Recent advances in elucidating the genetics of common variable immunodeficiency. Genes Dis.

[24] Rodriguez-Cortez VC, Del Pino-Molina L, Rodriguez-Ubreva J, Ciudad L, Gomez-Cabrero D, Company C (2015). Monozygotic twins discordant for common variable immunodeficiency reveal impaired DNA demethylation during naïve-to-memory B-cell transition. Nat Commun.

[25] Del Pino-Molina L, Rodriguez-Ubreva J, Torres Canizales J, Coronel-Diaz M, Kulis M, Martin-Subero JI (2019). Impaired CpG demethylation in common variable immunodeficiency associates with B cell phenotype and proliferation rate. Front Immunol.

[26] Rae W (2017). Indications to epigenetic dysfunction in the pathogenesis of common variable immunodeficiency. Arch Immunol Ther Exp.

[27] Martinez-Cano J, Campos-Sanchez E, Cobaleda C (2019). Epigenetic priming in immunodeficiencies. Front Cell Dev Biol.

[28] Babaha F, Yazdani R, Shahkarami S, Hamidi Esfahani Z, Abolhassani H, Sadr M (2021). Evaluation of miR-210 expression in common variable immunodeficiency: patients with unsolved genetic defect. Allergol Immunopathol.

[29] Seidel MG, Kindle G, Gathmann B, Quinti I, Buckland M, van Montfrans J (2019). The European Society for Immunodeficiencies (ESID) Registry working definitions for the clinical diagnosis of inborn errors of immunity. J Allergy Clin Immunol Pract.

[30] Luning Prak ET, Ross J, Sutter J, Sullivan K (2011). Age-related trends in pediatric B-cell subsets. Pediatr Dev Pathol.

[31] Yazdani R, Seify R, Ganjalikhani-Hakemi M, Abolhassani H, Eskandari N, Golsaz-Shirazi F (2017). Comparison of various classifications for patients with common variable immunodeficiency (CVID) using measurement of B-cell subsets. Allergol Immunopathol.

[32] Schatorje EJH, Gemen EFA, Driessen GJA, Leuvenink J, van Hout WNM, van der Burg M (2011). Age-matched reference values for B-lymphocyte subpopulations and CVID classifications in children. Scand J Immunol.

[33] Ogulur I, Kiykim A, Baser D, Karakoc-Aydiner E, Ozen A, Baris S (2020). Lymphocyte subset abnormalities in pediatric-onset common variable immunodeficiency. Int Arch Allergy Immunol.

[34] Piatosa B, Pac M, Siewiera K, Pietrucha B, Klaudel-Dreszler M, Heropolitanska-Pliszka E (2013). Common variable immunodeficiency in children- clinical characteristics varies depending on defect in peripheral B cell maturation. J Clin Immunol.

[35] Alkan G, Keles S, Reisli I (2018) Evaluation of clinical and immunological characteristics of children with common variable immunodeficiency. Int J Pediatr 3527480. 10.1155/2018/3527480 10.1155/2018/3527480 PMC5937368 29849668

[36] Szczawinska-Poplonyk A, Tapolska-Jozwiak K, Samara H, Boruczkowski M, Wieckowska B (2021). The CXCR5 T follicular helper cell compartment in children with antibody deficiencies- in search of a prognostic marker of childhood hypogammaglobulinemia. Allergol Immunopathol.

[37] Del Pino-Molina L, Lopez-Granados E, Lecrevisse Q, Torres Canizales J, Perez-Andres M, Blanco E (2021). Dissection of the pre-germinal center B-cell maturation pathway in common variable immunodeficiency based on standardized flow cytometric EuroFlow tools. Front Immunol.

[38] Lopez-Herrera G, Segura-Mendez NH, O’Farril-Romanillos P, Nunez-Nunez ME, Zarate-Hernandez MC, Mogica-Martinez D (2019). Low percentages of regulatory T cells in common variable immunodeficiency (CVID) patients with autoimmune diseases and its association with increased numbers of CD4+CD45RO+ T and CD21low B cells. Allergol Immunopathol.

[39] Kutukculer N, Azarsiz E, Aksu G, Karaca NE (2016). CD4+CD25+Foxp3+ T regulatory cells, Th1 (CCR5, IL-2, IFN-γ) and Th2 (CCR4, IL-4, IL-13) type chemokine receptors and intracellular cytokines in children with common variable immunodeficiency. Int J Immunopathol Pharmacol.

[40] Sanaei R, Rezaei N, Aghamohammadi A, Delbandi A, Teimourian S, Yazdani R (2019). Evaluation of the TLR negative regulatory network in CVID patients. Genes Immun.

[41] Kutukculer N, Azarsiz E, Karaca NE, Ulusoy E, Koturoglu G, Aksu G (2015). A Clinical and laboratory approach to the evaluation of innate immunity in pediatric CVID patients. Front Immunol.

[42] Zainaldain H, Sadaat Rizvi F, Rafiemanesh H, Alizadeh M, Jamee M, Mohammadi S (2020). Infectious complications reporting in common variable immunodeficiency: a systematic review and meta-analysis. Oman Med J.

[43] Huck K, Feyen O, Ghosh S, Beltz K, Bellert S, Niehues T (2009). Memory B cells in healthy and antibody deficient children. Clin Immunol.

[44] Berron-Ruiz L, Lopez-Herrera G, Vargas-Hernandez A, Mogica-Martinez D, Garcia-Latorre E, Blancas-Galicia L (2014). Lymphocytes and B cell abnormalities in patients with common variable immunodeficiency (CVID). Allergol Immunopathol.

[45] Lin L, Wang Y, Liu X (2015). Clinical and immunological features of common variable immunodeficiency in China. Chin Med J.

[46] Benavides-Nieto M, Mendez-Echevarria A, del Rosal T, Luz Garcia-Garcia M, Casas I, Pozo F (2019). The role of respiratory viruses in children with humoral immunodeficiency on immunoglobulin replacement therapy. Pediatr Pulmonol.

[47] Duraisingham SS, Manson A, Grigoriadou S, Buckland M, Tong CY, Longhurst HJ (2015). Immune deficiency: changing spectrum of pathogens. Clin Exp Immunol.

[48] Ramzi N, Jamee M, Bakhtiyari M, Rafiemanesh H, Zainaldain H, Tavakol M (2020). Bronchiectasis in common variable immunodeficiency: a systematic review and meta-analysis. Pediatr Pulmonol.

[49] Oksenhendler E, Gerard L, Fleischi C, Malphettes M, Mouillot G, Jaussaud R (2008). Infections in 252 patients with common variable immunodeficiency. Clin Infect Dis.

[50] Abolhassani H, Rezaei N, Mohammadinejad P, Mirminachi B, Hammarstrom L, Aghamohammadi A (2015). Important differences in the diagnostic spectrum of primary immunodeficiencies in adults versus children. Expert Rev Clin Immunol.

[51] Patel J, Kumar A, Agasti A, Choksey A, Phadke A, Sawant P (2012). CVID enteropathy- a rare cause of chronic diarrhea in a child. Indian J Pediatr.

[52] Borish L, Ayars AG, Kirkpatrick CH (2011). Common variable immunodeficiency presenting as herpes simplex virus encephalitis. J Allergy Clin Immunol.

[53] Aird A, Lagos M, Vargas-Hernandez A, Posey JE, Coban-Akdemir Z, Jhangiani S (2019). Novel heterozygous mutation in in NFKB2 is associated with early onset CVID and a functional defect in NK cells complicated by disseminated CMV infection and nephrotic syndrome. Front Pediatr.

[54] Abolhassani H, El-Sherbiny YM, Arumugakani G, Carter C, Richards S, Lawless S (2020). Expanding phenotype and novel insights into the pathogenesis of ICOS deficiency. J Clin Immunol.

[55] Asgardoon MH, Azizi G, Yazdani G, Sohani M, Pashangzadeh S, Kalantari A (2020). Monogenic primary immunodeficiency disorder associated with common variable immunodeficiency and autoimmunity. Int Arch Allergy Immunol.

[56] Azizi G, Abolhassani H, Mahdaviani SA, Chavoshzadeh Z, Eshghi P, Yazdani R (2017). Clinical, immunologic, molecular analyses and outcomes of Iranian patients with LRBA deficiency: a longitudinal study. Pediatr Allergy Immunol.

[57] Sun D, Heilmall J (2019). Disorders of CTLA expression, how they lead to CVID and dysregulated immune responses. Current Opin Allergy Clin Immunol.

[58] Azizi G, Ahmadi M, Abolhassani H, Yazdani R, Mohammadi H, Mirshafiey A (2016). Autoimmunity in primary antibody deficiency. Int Arch Allergy Immunol.

[59] Azizi G, Abolhassani H, Kiaee F, Tavakolinia N, Rafiemanesh H, Yazdani R (2018). Autoimmunity and its association with regulatory T cells and B cell subsets in patients with common variable immunodeficiency. Allergol Immunopathol.

[60] Matson EM, Abyazi M, Bell KA, Hayes KM, Maglione PJ (2021). B cell dysregulation in common variable immunodeficiency interstitial lung disease. Front Immunol.

[61] Feuille EJ, Anooshiravani N, Sullivan KE, Fuleihan RL, Cunningham-Rundless C (2018). Autoimmune cytopenias and associated conditions in CVID: a report from the USIDNET Registry. J Clin Immunol.

[62] Tillman R, Guillerman RP, Trojan T, Silva-Carmona M, Chinn IK (2019). Treatment-responsive granulomatous-lymphocytic interstitial lung disease in a pediatric case of common variable immunodeficiency. Front Pediatr.

[63] Patel S, Anzilotti C, Lucas M, Moore N, Chapel H (2019). Interstitial lung disease in patients with common variable immunodeficiency: several different pathologies?. Clin Exp Immunol.

[64] Jesenak M, Banovcin P, Jesenakova, Babusikova E (2014) Pulmonary manifestations of primary immunodeficiency disorders in children. Front Pediatr 2:77. 10.3389/fped.2014.00077 10.3389/fped.2014.00077 PMC4110629 25121077

[65] Najem CE, Springer J, Prayson R, Culver DA, Fernandez J, Tavee J (2018). Intra cranial granulomatous disease in common variable immunodeficiency: case series and review of the literature. Semin Arthritis Rheum.

[66] Weinberger T, Fuleihan R, Cunningham-Rundles C, Maglione P (2019). Factors beyond lack of antibody govern pulmonary complications in primary antibody deficiency. J Clin Immunol.

[67] Kebudi R, Kiykim A, Sahin MK (2019). Primary immunodeficiency and cancer in children: a review of the literature. Current Ped Rev.

[68] Piquer Gibert M, Alsina L, Giner Munoz MT, Cruz Martinez O, Ruiz Echevarria K, Dominguez O (2015). Non-Hodgkin lymphoma in pediatric patients with common variable immunodeficiency. Eur J Pediatr.

[69] Renzi S, Langenberg-Ververgaert KPS, Waespe N, Ali S, Bartram J, Michaeli O (2020). Primary immunodeficiencies and their associated risk of malignancies in children: an overview. Eur J Pediatr.

[70] Hauck F, Voss R, Urban C, Deidel M (2018) Intrinsic and extrinsic causes of malignancies in patients with primary immunodeficiency disorders. J Allergy Clin Immunol 141:59–68e4. 10.1016/j.jaci.2017.06.009 10.1016/j.jaci.2017.06.009 28669558

[71] Pittet LF, Postfay-Barbe KM (2021) Vaccination of immune compromised children- an overview for physicians. Eur J Pediatr epub ahead of print. 10.1007/s00431-021-03997-1 10.1007/s00431-021-03997-1 PMC8195953 33665677

[72] Shearer WT, Fleisher TA, Buckley RH, Ballas Z, Ballow M, Blaese M et al Recommendations for live viral and bacterial vaccines in immunodeficient patients and their close contacts. J Allergy Clin Immunol 2014;133:961–966. 10.1016/j.jaci.2013.11.043 10.1016/j.jaci.2013.11.043 PMC4009347 24582311

[73] Principi N, Esposito S (2014). Vaccine use in primary immunodeficiency disorders. Vaccine.

[74] Mieves JF, Wittke K, Freitag H, Volk HD, Scheidenbogen C, Hanitsch LG (2017). Influenza vaccination in patients with common variable immunodeficiency (CVID). Curr Allergy Asthma Rep.

[75] Baris S, Ercan H, Hasret Cagan H, Ozen A, Karakoc-Aydiner E, Ozdemir C (2011). Efficacy of intravenous immunoglobulin treatment in children with common variable immunodeficiency. J Investig Allergol Clin Immunol.

[76] Pandit C, Hsu P, van Asperen P, Mehr S (2016). Respiratory manifestations and management in children with common variable immunodeficiency. Pediatr Resp Rev.

[77] Gernez Y, Baker MG, Maglione PJ (2018). Humoral immunodeficiencies: conferred risk of infections and benefits of immunoglobulin replacement therapy. Transfusion.

[78] Papadopoulou-Alataki E, Hassan A, Davies G (2012). Prevention of infection in children and adolescents with primary immunodeficiency disorders. Asian Pac J Allergy Immunol.

[79] Van Stigt AC, Dik WA, Kamphuis LSJ, Smits BM, van Monfrans JM, van Hagen PM (2020). What works when treating granulomatous disease in genetically undefined CVID?. A systematic review Front Immunol.

[80] Romberg N, Lawrence MG (2019). Birds of a feather: common variable immunodeficiency. Ann Allergy Asthma Immunol.

